# Lipopolysaccharides: Regulated Biosynthesis and Structural Diversity

**DOI:** 10.3390/ijms24087498

**Published:** 2023-04-19

**Authors:** Satish Raina

**Affiliations:** Laboratory of Bacterial Genetics, Gdansk University of Technology, Narutowicza 11/12, 80-233 Gdansk, Poland; satish.raina@pg.edu.pl

The cell envelope of Gram-negative bacteria contains two distinct membranes, an inner (IM) and an outer (OM) membrane, separated by the periplasm, a hydrophilic compartment that includes a thin layer of peptidoglycan. The most distinguishing feature of such bacteria is the presence of an asymmetric OM with phospholipids located in the inner leaflet and lipopolysaccharides (LPSs) facing the outer leaflet. The maintenance of this OM asymmetry is essential to impart a permeability barrier, which prevents the entry of bulky toxic molecules, such as antibiotics and bile salts, into the cells [1]. LPS is a complex glycolipid that, with few exceptions, is essential for bacterial viability and is one of the major virulence factors in pathogenic Gram-negative bacteria. Model bacteria, such as *Escherichia coli*, contain approximately 2–3 × 10^6^ molecules of LPS that cover more than 75% of the OM [2]. The composition of LPS is highly heterogenous; however, they often share a common architecture, and for convenience can be divided into three parts. The first, a conserved glycophospholipid moiety called lipid A, which anchors LPS in the OM, constitutes the endotoxin principal since it is recognized by the innate immune cell receptor TLR4/MD2-CD14 complex. A proximal core oligosaccharide is attached to lipid A via 3-deoxy-α-D-*manno*-oct-2-ulsonic acid (Kdo), and in smooth bacteria a distal *O*-polysaccharide called an *O*-antigen is attached [3]. It should be noted that some bacteria display LPSs without the *O*-chain, which are thus named lipooligosaccharides (LOSs). The biosynthesis of LPS begins with the formation of the essential key precursor molecule Kdo_2_-lipid A, which requires the sequential action of seven essential enzymes on the cytoplasmic side of the IM. This Kdo_2_-lipid A serves as a substrate for an extension by the incorporation of various sugars by specific glycosyltransferases before the lipid A-core molecules are flipped by MsbA to the periplasmic side of the IM.

At the genetic and biochemical levels, the biosynthesis of LPS in bacteria such as *E*. *coli* has been well studied; however, it is not fully understood how amounts of LPS are regulated or how it is finally assembled in the OM. This is particularly important since bacteria must maintain a strict balance of 1:0.15 between phospholipids and LPSs, the two essential components of the cell envelope [4]. In *E*. *coli*, this is achieved by regulating the first committed step in LPS biosynthesis, which is catalyzed by the essential enzyme LpxC, and the activity of the FabZ dehydratase enzyme, which catalyzes the first step in phospholipid biosynthesis. This regulation is key to achieving a balance between phospholipid and LPS amounts since they use the same (*R*)-3-hydroxymyristate as the common metabolic precursor [5,6].

Thus, recent studies have highlighted the following: (i) The regulation of LPS assembly can unravel the intricate multilayered mechanisms regulating LpxC turnover in terms of demand for LPS synthesis. After the initial discovery that LpxC levels are controlled in a 20-fold range in relation to the lipid A content, without any increase in the corresponding mRNA levels of the encoded gene, FtsH was identified as the protease responsible for this regulation [5,7]. More recent studies have shown that FtsH-dependent proteolysis requires the essential IM-anchored LapB protein, and this can be counteracted by another essential protein, LapC, to regulate the quantity of LpxC [6,8,9,10,11,12]. New structural studies further elucidated the structure of LapC, showing that LapC binds to LPS, and further established an interaction of LapB with LapC and FtsH [13,14]. However, in one study, LapC binding to LPS in the periplasmic-exposed linker region was shown, and a role was proposed for the sensing of LPS accumulation in the IM, triggering LpxC degradation [13]. In another study, LapB binding to LPS or LapC in the IM transmembrane anchors was shown, and such interaction with either LPS or LapC was suggested to be mutually exclusive [14]. Although it is now established that LapB recruits FtsH for LpxC degradation and LapC acts as an anti-adaptor protein to restrict LpxC degradation, the precise sensing of LPS by LapC needs further elucidation. Since LapB co-purifies with LPS, FtsH, and several proteins involved in either LPS and phospholipid biosynthesis or LPS translocation, it was suggested that LapB could act as a hub to coordinate LPS and phospholipid synthesis and act as an assembly site [6]. This has recently gained further support based on an observed protein–protein interaction between LapB and FabZ, and LapB and lipid A biosynthetic enzymes (LpxA, LpxC, and LpxD), and LpxD was also found to be subjected to proteolytic control by LapB-FtsH [15]. Identifying the specific cellular signals that induce FtsH-LapB-mediated proteolysis is another emerging line of research and these may respond to the concentration or forms of acyl-ACPs or lipid A disaccharide, acyl-CoA levels, the activity of LpxK and FabI enzymes, the PldA phospholipase-dependent sensing of LPS in the OM, and the release of acyl chains by degrading phospholipids [5,16,17]. Since LpxC is not subjected to FtsH-mediated regulatory control in *Pseudomonas aeruginosa*, a recent finding showed that in this bacterium LpxC is activated by the peptidoglycan (PG) biosynthetic enzyme MurA, linking the biogenesis of LPS with PG synthesis [18]. (ii) Structural advances have been made in understanding the molecular basis of MsbA-mediated LPS transport and its selectivity towards hexaacylated lipid A species [19,20]. This has been further supported by the identification of several single amino acid substitutions that can potentially relax this specificity and facilitate the transport of penta- and tetraacylated lipid A [21,22]. (iii) A new role of cardiolipin synthase A in assisting the MsbA-mediated transport of LPS has also been recently described [22,23]. (iv) The molecular basis of structural alterations in the LPS that contribute to its heterogenous composition and how bacteria respond to defects in LPS biogenesis. In *E*. *coli* and some related bacteria, this is achieved by tightly regulated transcriptional and post-transcriptional control by the cell-envelope-responsive sigma factor RpoE, transcriptional factor RfaH, BasS/R, PhoP/Q, PhoB/R and Rcs two-component systems, as well as their non-coding arm invoking small regulatory RNAs, such as MicA, MgrR, RybB, and RirA [24,25,26,27]. Among the commonly observed non-stoichiometric modifications observed in lipid A are the incorporation of phosphoethanolamine (P-EtN), 4-amino-4-deoxy-L-arabinose (L-Ara4N), and LpxT-dependent phosphorylation at the 1′ position, the addition or removal of acyl chains, the modification of the inner core by the addition of a third Kdo residue, the decoration of Kdo and heptose I by P-EtN, the incorporation of rhamnose (Rha), uronic acid, and the truncation of the outer core [24,28]. However, some pathogenic bacteria also exhibit specific changes in the disaccharide backbone, lipid A acylation, and acyl chain lengths upon interaction with the host and changes in the host niche environment. Thus, in some bacteria, disaccharide backbones, with a 2, 3-diamino-2,3-dideoxy-D-glucose (DAG) backbone or with mixed compositions, have been identified [29]. Furthermore, defects in the composition and assembly of LPS activate the RpoE sigma factor and the CpxA/R two-component system, although initially it was thought that RpoE specifically monitors protein folding defects in the cell envelope [6,26,30]. (v) Finally, in the last decade, several studies have elaborated on the transport of LPS via the essential LPS transport system, comprising seven essential Lpt proteins which form a transenvelope apparatus with components residing in the cytoplasm, the IM, the periplasm, and the OM [31]. Some of these essential Lpt proteins have been targeted to develop antimicrobial peptides such as murepavadin (LptD) and antimicrobial compounds such as thanatin (LptC) [32].

Thus, this Special Issue presents eight manuscripts that cover the regulation of LpxC [21,33], the targeting of LpxC to design vaccines with reduced endotoxicity [34], structural diversity in the composition of LPS and non-stoichiometric modifications in lipid A [35,36,37,38], and the LPS-neutralizing activity of BPI proteins [39]. 

In a review article, Klein et al., provide an overview of the current knowledge on the regulation of LPS assembly and the cellular mechanisms by which bacteria employ a series of checkpoints to balance LPS and phospholipid biosynthesis [33]. The authors dwell on the regulation of availability of two critical early metabolic precursors: UDP-*N*-acetylglucosoamine (UDP-GlcNAc) and (*R*)-3-hydroxymyristoyl-ACP. The former is shared between peptidoglycan and LPS biosynthesis pathways, while (*R*)-3-hydroxymyristoyl-ACP is situated at another branch point since it is recruited for both phospholipid and lipid A biosynthesis [33]. The flux of (*R*)-3-hydroxymyristoyl-ACP in these two essential pathways is discussed in terms of the key regulatory control of the amounts of LpxC deacetylase, which mediates the first committed step in lipid A biosynthesis by FtsH protease in a complex with the LapB protein [5,6]. However, before 2020, it was unknown how the FtsH-LapB protease complex responds to the cellular demand for LPS to adjust LpxC amounts and hence the synthesis. Thus, the identification of LapC by five independent groups is covered and based on the structure of LapC and its interaction with LPS and LapB; it is suggested that LapC acts upstream of LapB-FtsH and counteracts FtsH-LapB acting as an anti-adaptor protein. The discovery of FtsH-independent degradation by HslVU protease and the negative regulation by GcvB sRNA is discussed, which can be used to further regulate the quantity of LpxC [8]. The additional function of LapB as a scaffold for LPS assembly based on the interaction with several LPS biosynthetic and transport proteins is further elaborated. Another important checkpoint mediated by the MsbA transporter, which ensures only the hexaacylated LPS with a complete core is delivered to the OM, is described. Recent MsbA structures in complex with LPS are discussed in the context of LPS recognition and selectivity for hexaacylated lipid A compared to tetraacylated species. Another checkpoint mediated by the RfaH transcriptional factor, which regulates the expression of the long *waa* operon by overcoming transcriptional termination, is also covered. The authors further describe how the RpoE sigma factor and various two-component systems regulate LPS heterogeneity, with RpoE-regulated RybB sRNA repressing the WaaR synthesis and providing another branch point in bacteria such as *E*. *coli* for the regulated synthesis of LPS with or without truncation in the outer core. Finally, the authors cover how the bacteria respond to defects in LPS by activating the transcription of the gene encoding the RpoE sigma factor. 

In the paper by Wieczorek et al., the authors report the discovery of LapD as a new partner of LapA/LapB proteins involved in the regulation of LpxC [21]. The inactivation of the *lapD* gene was shown to confer temperature sensitivity (Ts), sensitivity to antibiotics such as vancomycin, a reduction in LpxC amounts at elevated temperatures, a retention of a major portion of LPS in the IM, the synthetic lethality in the absence of acyltransferases (LpxL or LpxM), and cardiolipin synthase encoding gene *clsA* or MicA small regulatory RNA. Some of these phenotypic defects, such as vancomycin sensitivity or the conditional synthetic lethality of Δ(*lapD micA*), could be overcome when LpxC was stabilized due to mutations in either *lpxC*, *lapB*, or *ftsH* genes, mimicking the properties of strains lacking the C-terminal domain of the essential LapC protein. Consistent with a role of LapD in the regulation of LpxC and the trafficking of LPS, mutations that lower the amounts of lipid A synthesis were lethal in the absence of LapD and suppressors mapping to the *msbA* gene encoding LPS flippase can bypass the lethality of either Δ(*lpxL lapD*) or Δ(*lpxM lapD*). Thus, the authors proposed that LapD could be upstream of the LapB-FtsH complex in the regulation of the amount of LpxC. Consistent with such a notion, the authors show that the overexpression of genes such as *srrA* (encoding a putative transcription factor) can suppress the Ts phenotype of a Δ*lapD* strain with a concomitant restoration of LpxC levels. Taken together, the authors suggest that LapD is part of the LapB complex and acts as an antagonist of LpxC degradation by the LapB-FtsH complex.

The Gram-negative bacterium *Bordetella pertussis* produces LPSs lacking *O*-antigens. *B. pertussis* is the causative agent of a respiratory infection known as whooping cough, which in the past has been controlled using whole-cell pertussis vaccines (wP); however, due to the reactogenicity of wP and its endotoxin content, new vaccines are being sought. The manuscript by Pérez-Ortega et al., describes a new strategy to reduce the endotoxicity of whole-cell outer membrane vesicle (OMV)-based vaccines against this bacterium [34]. Thus, the authors used the regulated expression of the *lpxC* gene to reduce the amounts of LPS. Such a constructed strain was found to elicit a reduced Toll-like receptor 4 (TLR4)-stimulating activity. Interestingly, the authors show that the LpxC-depleted strain resulted in a significant increase in OMV production, and such a strain showed a reduction in the LPS content in OMVs and hence could be used to develop OMV-based vaccines.

As the structural diversity of lipopolysaccharides contributes towards bacterial adaptation in diverse environments and the host–pathogen interaction, and is a critical virulence factor in pathogenic Gram-negative bacteria, in this Special Issue these aspects are addressed. In one manuscript, the authors examined the structure of LPS from *Liberibacter crescens* (Lcr), which is closely related to *Candidatus* Liberibacter asiaticus, the causative agent of citrus greening disease [35]. A combination of mass spectrometric and NMR analyses showed that its major lipid A species consists of a pentaacylated 6-linked GlcN disaccharide lacking phosphate residues. However, lipid A species containing a single phosphate group were also observed. The authors further show that two of the three *O*-polysaccharide structures comprise repeating ribofuranose and galactose moieties instead of deoxyhexose residues. Finally, it is proposed that the reduced *O*-polysaccharide size observed for Lcr bacteria could be important for interactions with its host.

Another manuscript deals with the regulated remodeling of lipid A in plant pathogenic bacteria *Pseudomonas syringae* pv. *phaseolicola* (*Pph*) 1448A [36]. The authors first performed a proteomic search to identify the orthologous genes *pagL*, l*pxO*, and *eptA*, whose products are predicted to be involved in lipid A modifications. PagL hydrolyzes an ester-linked acyl chain at the O-3 position of the distal glucosamine of lipid A and LpxO catalyzes the hydroxylation of secondary acyl chains in *P. aeruginosa* [36]. EptA is known to mediate P-EtN transfer at the 1-phosphate of lipid A [24,25]. Next, the authors constructed isogenic null strains lacking these genes and analyzed their lipid A, showing that in the absence of LpxO, it lacks hydroxylation of secondary fatty acids. Similarly, the function of PagL-mediated deacylation was found to be conserved, and P-EtN modifications of lipid A were absent in the absence of EptA. Curiously, the authors did not observe lipid A modification with L-Ara4N, although a homolog of ArnT was identified based on proteome analysis. Overall, lipid A modifications observed in isogenic deletion derivatives can be further used to study the role of lipid A modifications during pathogen–plant interactions. It is pertinent to also point out that some of these modifications, such as P-EtN addition, confer resistance against cationic antimicrobial peptides, are critical for maintaining the integrity of the outer membrane, and PagL-mediated deacylation can impact OMV production [2,36].

Two studies address specific LPS structural alterations in the plant pathogenic bacterium *Pectobacterium parmentieri* and the human pathogen *Proteus mirabilis* [37,38]. In the study on *P. parmentieri*, the heterogeneity of the *O*-polysaccharides (OPS) of two strains was examined by chemical composition analysis and NMR spectroscopy. The authors show that the OPS of these strains consist of → 3)-Gal*f*, → 3)-Gal*p*, two residues of → 6)-Glc*p*, and a rare residue of 5,7-diamino-3,5,7,9-tetradeoxy-L-*glycero*-L-*manno*-non-2-ulosonic acid substituted in the position C-8 [37]. In the case of *P. mirabilis*, the study describes the serological properties for two smooth strains, showing that their *O*-antigens are unique and represent a new serotype O-84 [38]. Thus, the authors analyzed the OPS composition by chemical and NMR spectroscopy. These experiments revealed that their N-acetylglucosamine (GlcNAc) residues are non-stoichiometrically *O*-acetylated at positions 3, 4, and 6, or 3 and 6, and a minority of α-GlcNAc residues are 6-*O*-acetylated. Overall, some of these studies on LPS heterogeneity reveal that structural alterations can exist in all three parts of LPS and can contribute to pathogenicity and adaptation to different environments [36,37,38].

It is well established that the causative agent of Gram-negative bacteria is LPS. Sepsis is often a life-threatening disease and a major cause of mortality in neonates. Thus, it is imperative to gain information on the genetic variations associated with genes whose products can impact the susceptibility or risk associated with sepsis. In this direction, Ederer et al., examined the polymorphism of the neutrophil-derived bactericidal/permeability-increasing protein encoding the BPI gene, since BPI exhibits bactericidal activity towards Gram-negative bacteria and is known for its potent anti-inflammatory and LPS-neutralizing activity [39]. The authors addressed the effect of changes in the amino acid at amino acid position 216, since structural examination predicts that the residue is surface-exposed and proximal to the lipid-binding pocket in the N-terminal domain of BPI. Thus, two variants, BPI_216K_ (with positively charged lysine) and BPI_216E_ (with negatively charged glutamic acid), were examined for their interaction with LPS and bactericidal activity. Significantly, this study shows that by stimulating human peripheral blood mononuclear cells, BPI_216K_ exhibits a superior LPS-neutralizing capacity compared to BPI_216E_ in respect to IL-6 secretion. Thus, the authors provide a rational explanation for a favorable outcome of sepsis in the presence of BPI_216K_ and the decreased LPS-neutralizing capacity of BPI_216E_. These results can further help in evaluating the administration of BPI during Gram-negative sepsis.
